# A New, Quick Method for Testing Organic Soils Based on the Electrical Impedance Spectrum of the Measuring Coil

**DOI:** 10.3390/ma19020381

**Published:** 2026-01-17

**Authors:** Barbara Solecka, Andrzej Nowrot, Katarzyna Nowińska, Jarosław Sikorski, Adam Michczyński

**Affiliations:** 1 Institute of Physics—Centre for Science and Education, Silesian University of Technology, Konarskiego 22B, 44-100 Gliwice, Poland; jaroslaw.sikorski@polsl.pl (J.S.); adam.michczynski@polsl.pl (A.M.); 2 Department of Electrical Engineering and Automation in Industry, Silesian University of Technology, Akademicka 2, 44-100 Gliwice, Poland; andrzej.nowrot@polsl.pl; 3 Department of Applied Geology, Silesian University of Technology, Akademicka 2, 44-100 Gliwice, Poland; katarzyna.nowinska@polsl.pl

**Keywords:** impedance spectroscopy, organic soil, environmental changes

## Abstract

This paper presents a new, quick method for testing the content of magnetic forms of iron in organic soils. These forms are an important marker of changes occurring in the environment. This method is based on impedance spectroscopy of a measuring coil inside which the tested material is placed—the material serves as the core of the coil. Unlike EIS (electrochemical impedance spectroscopy), the new method does not use electrodes, is sensitive to magnetic forms of iron, and is non-contact (the measuring current does not flow through the tested material). The results of research on three materials, including brown peat and silt with plant detritus, are presented in this paper. The results showed that changes in the standardized components of the measuring coil impedance in the frequency range of 100–135 kHz enable the determination of the content of ferromagnetic iron oxide (Fe_3_O_4_). The proposed method is very sensitive to soil oxide content in the range of 0% to 8%. Additionally, elemental composition analysis was performed using ICP-AES (inductively coupled plasma–atomic emission spectroscopy), which allowed for comparison of iron and other metal content with impedance measurement results. The final results confirm the usefulness of impedance spectroscopy as a non-destructive method for studying sedimentary environments and assessing their mineral properties.

## 1. Introduction

The state of the natural environment in the past can be reconstructed based on the physicochemical properties of sediments and soils, which preserve traces of geochemical and mineralogical processes. One of the parameters widely used in such analyses is magnetic susceptibility. It allows for the assessment of the content of magnetic iron minerals in sedimentary material [1,2,3,4]. In natural soils and sediments, iron oxides and hydroxides are formed as a result of the weathering of parent rocks and subsequent diagenetic changes.

The most important are magnetite (Fe_3_O_4_), hematite (Fe_2_O_3_), goethite (α-FeOOH), and limonite (FeOOH·nH_2_O). These minerals play a crucial role in soil-forming processes and the biogeochemical cycle. Their presence influences the physical and chemical properties of sediments, such as color, sorption capacity, electrical conductivity, and nutrient availability for plants. The Fe^2+^ and Fe^3+^ ionic forms of iron are crucial for redox processes that determine the mineral and biological conditions of peatlands and river environments. Furthermore, these ions determine iron bioavailability and influence metabolic processes, including photosynthesis [5,6].

Peats and organic soils containing silt with plant detritus are a specific category of soil environment. Peatlands are characterized by a high content of organic matter, and their chemical and physical properties are largely dependent on the degree of humification and water conditions. Peats and organic muds can be considered important archives of natural environmental processes. Peat accumulates in conditions of limited oxygen and high humidity, which favors the preservation of organic material and the accumulation of iron compounds. Organic silts, rich in plant detritus, form in marsh and lake environments. Their composition reflects both sedimentation processes and local redox conditions [7,8,9].

Studying the magnetic properties of these types of materials provides valuable information about the course of past natural processes of weathering, mineralization, and accumulation of elements.

Research results on soil magnetic susceptibility in the global literature to date have focused primarily on areas exposed to industrial emissions. Thompson and Oldfield [1] presented the basics of so-called environmental magnetism, indicating that the analysis of magnetic signals can be used to reconstruct the history of pollution and natural processes.

In the paper [10] by Chaddha and Seehra, it was shown that dusts from coal combustion contain magnetic fractions of various grain sizes, which can be identified by magnetic susceptibility measurements. Hanesch and Scholger [11] used that method to map heavy metal contamination in soils, which confirmed its usefulness in assessing environmental anthropopression. At the same time, measurement methods were developed—from classical weight measurements and magnetometry sensitive to weak magnetic signals to frequency analyses of magnetic susceptibility allowing the distinction of grain fractions and their genesis [1,11].

A significant contribution to the development of analytical methods was also made by Förster [12,13], who proposed the concept of effective permeability and normalized impedance components, enabling precise characterization of magnetic materials. Bozorth’s classical studies [14] on ferromagnetism provided the foundation for further research on the magnetic properties of iron compounds.

In recent decades, magnetic methods have also been developed for engineering and diagnostic applications, including stress detection and monitoring of fatigue processes in steel elements [15,16,17]. Despite the above methods providing fundamental knowledge of magnetic processes in soils, they require specialized equipment, complex analytical procedures, and do not always allow for a clear connection between magnetic properties and the chemical composition of samples. However, the research by Żurek et al. [17] confirmed the usefulness of impedance spectroscopy as a non-destructive method in material analyses. In [18], the authors discuss the detection of soil density changes using a flat spiral coil and impedance spectroscopy. It should be noted that testing with such a coil has limited possibilities (the tested material is not the core of the coil, but adheres to it on one side). This is one of the very few examples of a measurement system based on coil impedance spectroscopy. Another application of impedance spectroscopy in soil research is presented in [19], but the authors use the traditional configuration of impedance measurement setup (without a measurement coil).

In the present work, we propose applying modified impedance spectroscopy as a method allowing for the simultaneous assessment of magnetic and electrical parameters of organic soils. Typically, impedance spectroscopy involves contact measurement of the electrical parameters of the material being tested (measuring electrodes are inserted into the tested material, or the tested preparation is placed in a measuring cuvette with electrodes on the walls).

In the modified method presented in this paper, a test tube containing the organic soils to be tested is placed inside a measuring coil. As a result, the material affects its coil impedance spectrum. It should be noted that the tested material does not have any contact with electrical components (e.g., electrodes). Because no measurement current flows through the soil being tested and there are no electrodes, no unfavorable electrode-related phenomena occur. This makes interpretation of measurement results easier. Moreover, the tested material is in the form of loose granules (not a solid material) and typically has a metal content of no more than a few percent. This prevents the alternating magnetic field of the measuring coil from inducing eddy currents in the tested material. The proposed technique, using reference materials, allows for effective measurement of the concentration of the ferromagnetic component of soil. Unfortunately, multiple competing mechanisms occur simultaneously within the sample, making the interpretation of the measured magnetic permeability very difficult. However, this interpretation is not necessary; it is enough to calibrate the system using reference material.

The above new technique allows for the determination of the content of ferromagnetic iron compounds, primarily Fe_3_O_4_, by analyzing changes in the impedance spectrum of the measuring coil (and, indirectly, magnetic permeability as a function of frequency). Thus, the magnetite content, unlike other iron oxides (which have a minor effect on magnetic properties), significantly affects the magnetic permeability and electrical conductivity of the samples. Additionally, in this paper, inductively coupled plasma atomic emission spectroscopy (ICP-AES) was used to determine the total content of iron and other elements.

Combining these methods allows for the search for correlations between the physical and chemical properties of the studied samples. This may contribute to a better understanding of the natural processes of iron mineralization and transformation in organic environments.

## 2. Methods

Impedance spectroscopy is a well-known technique that has been used for decades. The principle of operation of this technique is based on the flow of a sinusoidal alternating electric current with a programmable changing frequency through the tested material.

Simultaneously, the voltage at the sample terminals, the current in the sample, and the phase shift angle between the voltage and current waveforms are measured. Based on this, the measuring device (usually an LCR impedance bridge) indicates (calculates) the complex impedance of the tested object.

During impedance spectroscopy testing, impedance and phase are recorded as the frequency is swept over a wide range. Thanks to this, the real and imaginary components of the impedance are calculated, allowing the Nyquist curve to be plotted.

It should be noted that the Nyquist impedance curve is unique to each object. This provides an opportunity to identify the material being tested or detect its components.

The impedance spectroscopy testing procedure depends on the form of the material being tested. If the tested material is powdered, then it is placed in a measuring cuvette with electrodes on opposite walls. Sometimes, for powdered material, electrodes are driven into it (similar to soil resistivity testing methods). For a solid sample (e.g., in the form of a compressed pellet or rock), selected walls are painted with silver or copper paint, and wires are glued to them—this is how electrodes are created.

The application area of impedance spectroscopy is very wide. It covers not only the testing of materials but also the testing of manufactured components, e.g., electrochemical cells [20,21] and photovoltaic cells [22,23]. In [24], the authors used impedance spectroscopy for interdisciplinary research to detect Huanglongbing Bacterium. Another area of application is the testing of crack detection in aluminum plates for the aerospace industry [25]. Impedance spectroscopy has enormous application potential, but so far it has not been used to study organic soil using a measuring coil. The presented paper is the first study of organic soil using that method. A detailed description of impedance spectroscopy, exemplary research results, and application possibilities are described in [26,27,28]. In [29], the authors present an interesting application of impedance spectroscopy for the quantitation of total soil carbon (TSC), and in [30], they present the same for dynamic tracking of soil organic matter. Impedance spectroscopy is also used to investigate soil health [31].

It should be noted that in impedance spectroscopy, a very low current flows through the tested sample (typically microamps or milliamperes between electrodes). The magnetic field generated by this current is characterized by a very low magnetic flux density (negligibly low) and does not cause any noticeable magnetic interactions with the tested material. Therefore, the above standard method of applying impedance spectroscopy does not allow the detection of the ferromagnetic component of the tested material.

A different and completely new approach to impedance spectroscopy is presented in this paper. The use of a measuring coil in combination with impedance spectroscopy opens up new possibilities. The measuring coil generates a magnetic field strong enough to detect ferromagnetic components in the material sample. In the proposed method, the soil sample is the core of the measuring coil.

**Figure 1:**
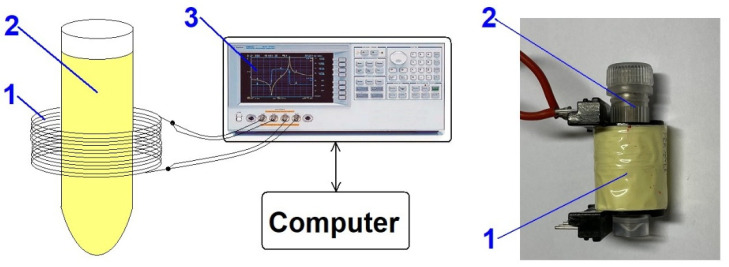
Measurement system. 1—Measuring coil; 2—test tube with the tested material; and 3—Agilent 4294A impedance analyzer.

The spectral characteristic of the coil impedance depends on the magnetic permeability, electrical conductivity, and grain size of the tested material. Introducing the tested material into the measuring coil changes the Nyquist curve. Figure 1 shows the measurement system. In that application, the coil is connected to the Agilent (Santa Clara, CA, USA) 4294A impedance analyzer.

The analyzer allows for measuring impedance in a frequency range from 40 Hz to 110 MHz. To ensure accurate measurements with this device, calibration and compensation were performed as standard. After calibration, the instrument’s measurement accuracy ranged from 0.03% to 0.08% (as per technical data and catalog values). To statistically assess external factors affecting coil performance, three measurement series were conducted. In each series, the analyzer measured each measurement point 16 times (iteration). Statistical analysis allowed us to estimate the resistance uncertainty (u(R)) to be 0.2% and the reactance uncertainty (u(L)) to be 0.4% (where L denotes inductance, and R denotes resistance in a serial RL circuit). The measuring coil used was 40 mm long, 10 mm in inner diameter, 18 mm in outer diameter, and had a resonant frequency of approximately 130 kHz. The investigated material was placed over a distance of 20 mm centrally and symmetrically inside the coil (the length of the test tube housing was 70 mm, but the material was located only in its central section). The inner diameter of the test tube was 9 mm.

The complex impedance of an empty coil in the series equivalent circuit is given by Equation (1) [32]. It should be noted that the series R-L model of the coil becomes less accurate as the frequency increases. This is caused by an increase in electrical capacitance between the coil turns and the skin effect in the coil wire as the frequency increases. These are the main reasons why in the serial R-L model, the real component of the impedance of the empty coil is identical to its resistance only at low frequencies. Similarly, the imaginary component of the impedance of the empty coil is identical to the inductive reactance ωL_0_ only at low frequencies. Placing a core inside a coil changes its impedance components (2) [32]. This is primarily manifested by an increase in inductance.

(1) $$Z_{0}=R_{0}+j\omega L_{0}$$

(2) Z=R+jωL

where

Z_0_ denotes the complex impedance of the empty measuring coil;
Z denotes the complex impedance of the measuring coil with the investigated material as a core;
R_0_ denotes the real component of the empty coil impedance (R_0_ becomes the coil resistance at low frequency);
R denotes the real component of the coil impedance with the tested material as the core (R → R_0_ at low frequency);
L_0_ denotes the inductance of the empty measuring coil in a series equivalent RL circuit;
L denotes the inductance of the measuring coil with the tested material inside as the core (in a series equivalent RL circuit);
ω denotes the angular frequency.

Using impedance spectroscopy methods, it is possible to analyze the state of the tested material by examining its relationships with measurable electrical and magnetic quantities.

One of the most important parameters describing the magnetic properties of materials is magnetic permeability. In fact, the magnetic permeability of any material depends on the alternating field frequency and is a complex function in the frequency domain. The imaginary component of that complex function is the result of the magnetic hysteresis phenomenon.

Changes in complex magnetic permeability can be indirectly studied by observing changes in the impedance of the coil with the tested material inside. Effective magnetic permeability is a complex quantity characterizing the state of the tested material in an alternating magnetic field. This quantity depends on the sample dimensions (especially when at least one of the dimensions is small or the material is grainy), the specific conductivity, the magnetic permeability of the solid material, and the frequency of the external magnetic field.

Based on the considerations proposed by Förster and on the applications described in [12,13,16], the concept of effective permittivity μ_eff_ is introduced in Equation (3). The following approximate equation is true if we neglect the real component of the impedance of the empty measuring coil (it is much smaller than the imaginary component).

(3) $$\frac{Z}{\omega L_{o}}\approx j\left(1-\eta +\eta \mu_{r}\mu_{eff}\right)$$

(4) $$\eta ={\left(\frac{D_{sample}}{D_{coil}}\right)}^{2}$$

where

µ_r_ denotes the relative magnetic permeability;
µ_eff_ denotes the effective magnetic permeability (inter alia, it depends on the specific conductivity);
η denotes the coil fill factor;
D_sample_ denotes the diameter of the sample with the investigated material;
D_coil_ denotes the equivalent coil diameter (the empty real coil with multiple winding layers and inductance L_0_ is replaced by a theoretical coil with a single winding layer and the same inductance, but with a consequently new diameter D_coil_).

Although introducing the tested material into the measuring coil causes a change in the Nyquist curve, these changes may sometimes be too weak to precisely determine the ferromagnetic content of the sample. F. Förster proposed the creation of two new functions in the angular frequency domain for all measurement bands [16] (noted in this paper as h_1_ and h_2_ in Equations (5) and (6)). F. Förster carried out these considerations for the R-L series model of the equivalent coil. The function h_2_(ω) can be considered as the normalized imaginary component of the coil impedance.

(5) $$h_{1}(\omega )=\frac{R-R_{0}}{\omega L_{0}}=\frac{\Delta R}{\omega L_{0}}=\eta \mu_{r}(-Im{\{\mu }_{ef}\})$$

(6) $$h_{2}(\omega )=\frac{\omega L}{\omega L_{0}}=1-\eta +\eta \mu_{r}Re{\{\mu }_{ef}\}$$

The important advantage of functions h_1_(ω) and h_2_(ω) is their high sensitivity to changes in the coil impedance spectrum. That sensitivity is clearly greater than the changes observed in the Nyquist image. Therefore, the solution proposed by F. Förster is very useful for research involving measuring coils and is used in this paper. It should be noted that the object of investigation is the organic soil inside the coil, not the measuring coil. Therefore, the complexity of phenomena in the measuring coil is not crucial. The measuring coil is always calibrated before starting the experiments, and the spectral characteristics of the empty coil are stored in the computer’s memory.

Additionally, the elemental composition of samples S1, S2, and S3 was measured using the well-known inductively coupled plasma spectrometer ICP-AES with Horiba JY 2000 (Jobin Yvon Horiba, Paris, France).

The above-mentioned spectrometer is an instrument used for chemical analysis, specifically for determining the elemental composition of various samples. It uses inductively generated plasma to ionize sample atoms and then analyzes the emitted electromagnetic radiation. Determining the elemental content of a sample involves measuring the intensity of radiation emitted at the individual spectra for that element. All atoms and ions in an excited state can simultaneously emit their characteristic radiation, making the method highly selective.

## 3. Materials

Iron oxides and hydroxides constitute a very important group of minerals in soil. These compounds play a crucial role in soil-forming processes as natural sorbents and influence the availability of plant nutrients such as phosphorus. They are particularly important for soil-forming processes. The Fe^2+^ and Fe^3+^ forms of iron found in soil affect the absorption of this element (iron) by plants. Iron deficiency can lead to impaired photosynthesis and cause chlorosis in plants. The main representatives of this group are magnetite (Fe_3_O_4_), hematite (Fe_2_O_3_), goethite (α-FeOOH), and limonite (FeOOH·nH_2_O). The investigated materials were collected in south-western Poland, in the Lower Silesia region, in the vicinity of the village of Nimil. The geographical coordinates of the sampling site are 50°51′21″ N and 17°14′51″ E (Greenwich reference). The sampling area is located within the Oława River valley. All samples were taken from the same location at different depths below ground level. The material was collected as a continuous core using an Instrof probe (Table 1). All test samples were dried at a uniform temperature, then ground in a mortar and sieved. The samples were free-flowing and uniformly sized.

## 4. Results and Discussion

To correctly analyze the tested soil, the measuring system must be calibrated using an empty coil (thereby obtaining background characteristics) and reference materials. Therefore, a sample of clean sand and a mixture of sand and a ferromagnetic pure Fe_3_O_4_ compound were prepared. These prepared sand samples (reference materials) contained between 0.4% and 8% Fe_3_O_4_ (iron oxide). This content range is intentional—typical iron content in soil is between 0.2% and 5%, although in some soils the content can be as high as 55%. The procedure for preparing reference materials is described in [33]. The prepared test material (the investigated material), in the form of compressed powders of the selected soils, was placed in a suitable cuvette inside the measuring coil. The measuring coil was connected to the precision impedance analyzer, Agilent 4294A, as shown in Figure 1. Measurements were carried out in a frequency range from 40 Hz to 200 kHz.

**Figure 2:**
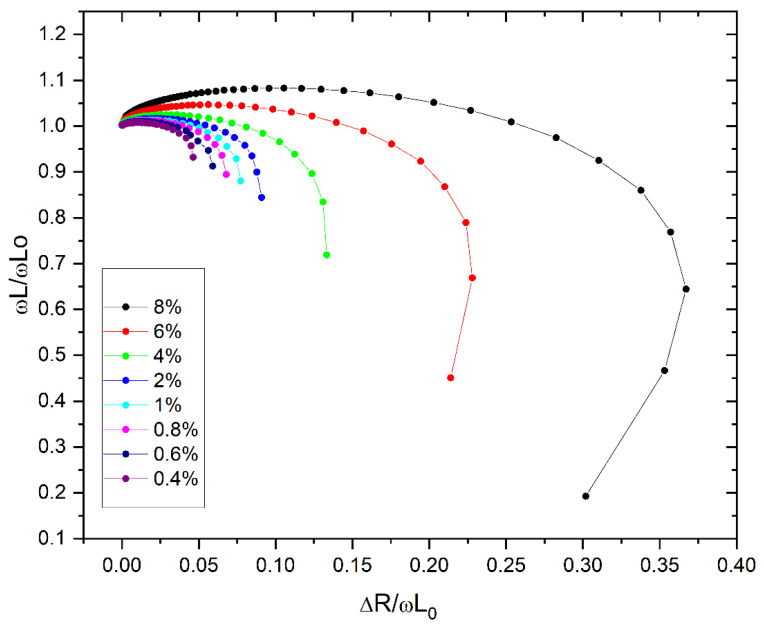
Characteristics of normalized impedance components based on Equations (5) and (6). Results obtained for reference materials with Fe_3_O_4_ concentrations ranging from 0.4% to 8%.

The characteristics, shown in Figure 2, are obtained in three calculation steps. During measurements, the impedance analyzer records the coil’s impedance and phase within a set frequency band. The analyzer’s internal computer then automatically calculates the resistance and inductance for each frequency in the R-L series equivalent circuit. These results are then loaded onto the PC. The above procedure is performed twice: once for the empty coil and once for the coil with the tested material.

According to Equations (5) and (6), the values of functions h_1_(ω) and h_2_(ω) are determined for each measurement frequency, as shown in Figure 2. Individual points on the same curve correspond to different angular frequencies.

**Figure 3:**
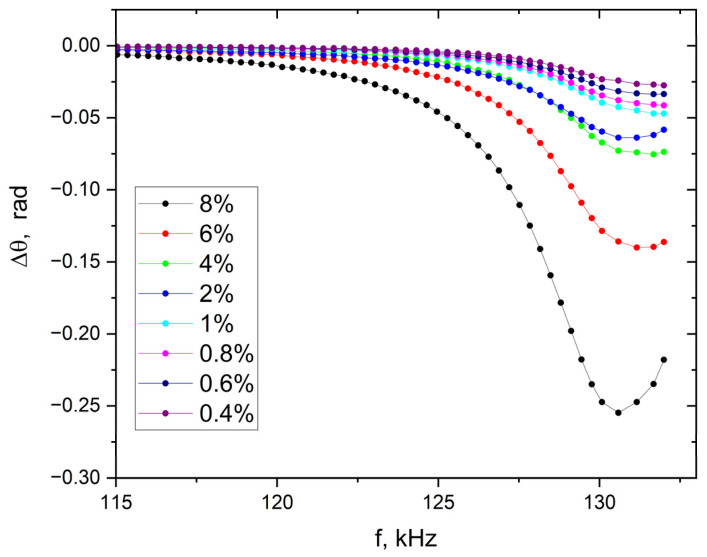
Spectral characteristics of the phase difference in the impedances according to Equation (9). Results obtained for reference materials with Fe_3_O_4_ concentrations ranging from 0.4% to 8%.

An equally sensitive method for changes in the Fe_3_O_4_ content in the tested material is the analysis of the spectral characteristics of the phase difference (Equation (9)) for the impedance of the coil with the sample (Equation (8)) and the impedance phase of the empty coil (Equation (7)) [32]. The obtained spectral characteristics of the phase difference in the impedances are shown in Figure 3. The highest sensitivity of the method was obtained for the frequency band ranging from 115 kHz to 135 kHz. This particular band covers the resonant frequency of the measuring coil. No similar changes were observed below 110 kHz and above 150 kHz.

(7) $$\theta_{0}\left(\omega \right)=arc\;tan\frac{ImZ_{0}\left(\omega \right)}{ReZ_{0}\left(\omega \right)}$$

(8) $$\theta \left(\omega \right)=arc\;tan\frac{ImZ\left(\omega \right)}{ReZ\left(\omega \right)}$$

(9) $$∆\theta (\omega )=\theta (\omega )-\theta_{0}(\omega )$$

where

Ѳ_0_(ω) denotes the spectral characteristics of the impedance phase of the empty coil;
Ѳ(ω) denotes the spectral characteristics of the impedance phase of the coil with the tested material;
∆Ѳ(ω) denotes the spectral characteristics of the phase difference in the impedances.

The resonance of any coil is the result of its inductance and the electrical capacitance that arises between the windings. Contrary to appearances, the equivalent circuit of a real coil is very complicated. Only approximate replacement schemes are used in practice. When sweeping the frequency during a coil impedance measurement, the impedance analyzer can show a sudden, almost abrupt change in impedance phase as the frequency passes through the resonance. This phenomenon is extremely violent. Suddenly, as the frequency increases, the impedance phase changes from positive to negative. A strong, but not as abrupt as in-phase, change in impedance modulus is also observed. Since coil resonance is a sudden phenomenon, even a small change in coil inductance can cause the coil state to jump from one side to the other around the resonance peak (this causes easily measurable phase changes). Therefore, if measurements are made near resonance, the system will be extremely sensitive to even the smallest changes in inductance. If a core (the material being tested) inside the coil is placed, it affects the coil’s inductance, and any changes in this inductance are easily detected when operating near the resonance. This is the primary reason for working near resonance.

The charts presented in Figure 2 and Figure 3 clearly indicate that the proposed methods based on impedance spectroscopy are very sensitive to the ferromagnetic form of iron oxide. The highest sensitivity of the proposed methods occurs at a frequency of 130.4 kHz.

**Figure 4:**
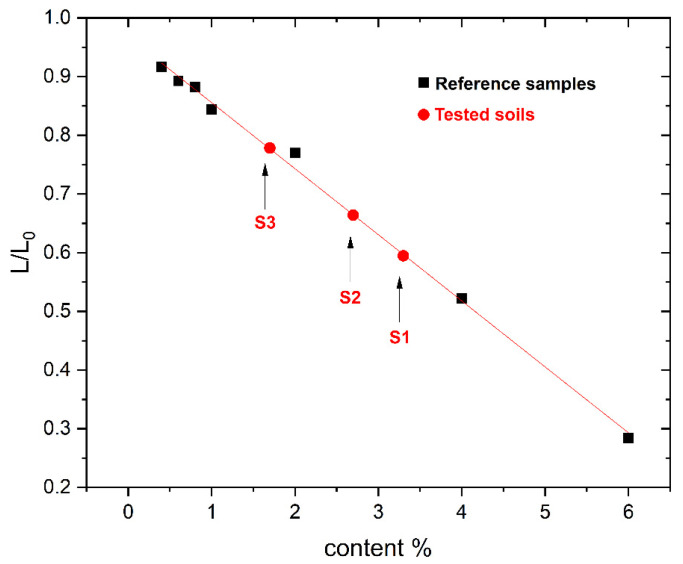
Normalized impedance imaginary component as a function of the content of Fe_3_O_4_ at a frequency of 130.4 kHz. The approximating line was determined from the points of the reference samples (black squares).

Therefore, based on the results in Figure 2, the ωL/ωL_0_ ratio is calculated for each reference sample concentration at an angular frequency of ≈819∙10^3^ rad/s (2∙π∙130.4∙10^3^ rad/s). As a result, the dependence of the normalized impedance imaginary component as a function of Fe_3_O_4_ concentration is determined (Figure 4). An approximating line is fitted (least squares method) to the measurement points for the reference materials—(Equation (10)). Based on that equation, the concentration of ferromagnetic iron in the tested soil samples is determined (Table 2).

(10) $$\frac{L}{L_{0}}=A\cdot c+B$$

where

c denotes the content of Fe_3_O_4_ in %;
A denotes the sensitivity coefficient, A = −0.1126(28) $\frac{1}{\%}$;
B denotes the equation coefficient, offset, B = 0.968(8).

The impedance response of the measuring coil reflects not only the content of ferromagnetic minerals but also natural environmental variability, including grain size distribution, organic matter content, and the electrical conductivity of the soil matrix. In organic soils, moisture affects the measurements indirectly by modifying conductivity and effective magnetic permeability; however, the absence of direct electrical contact between the sample and the measuring system reduces sensitivity to electrode-related moisture artifacts.

ICP-AES studies were carried out simultaneously. Table 3 shows the concentration of selected elements in the studied samples. The dried and ground samples (fraction size: 0.1 mm) were mineralised using a UniClever microwave mineraliser (Plazmatronika, Wrocław, Poland) at a 1:4 ratio of a 65% solution of nitric acid (HNO_3_) and a 37% solution of hydrochloric acid (HCl). This process was repeated twice for each sample for replication purposes. The prepared solutions were analyzed for Cr, Al, Ni, Cu, Zn, Fe, Ba, Mg, Mn, Ti, and Zr using a JY 2000—Sequential ICP-AES spectrometer (Jobin Yvon Horiba, Paris, France). The repeatability of the measurements was verified by repeating the preparation and measurement process twice for each sample. Additional analyses of the samples were performed to verify the calibration of the instrument. All the above values are far above the lower detection limit.

Particular attention should be paid to the iron content obtained from ICP-AES measurements. For all samples, the iron content in Table 3 is lower compared to the Fe_3_O_4_ content in Table 2.

It should be noted that the ICP-AES technique provides information on the elemental composition of the tested material, not on the chemical compounds contained in it. This means that the ICP-AES method cannot distinguish between the ferromagnetic Fe_3_O_4_ and paramagnetic Fe_2_O_3_ forms.

However, the method proposed in this paper, based on coil impedance spectroscopy, is selective and detects only the ferromagnetic form. A comparison of the results obtained using both methods is presented in Table 4.

The discrepancy between the ICP-OES and impedance spectroscopy results is not surprising. The ratio of contents in Table 4 covers a range from 1.29 (Sample S3) to 1.35 (Sample S1). ICP-AES measures the percentage of iron as an element, while impedance spectroscopy measures the percentage of a chemical compound. Consider the molecule Fe_3_O_4_: the spectrometer ICP-AES detects three Fe atoms in the molecule. However, impedance spectroscopy detects the ferromagnetic molecule Fe_3_O_4_, which consists of three Fe atoms and four O atoms (not exclusively Fe atoms). The above consideration is presented in Table 5. The calculation results show that the Fe_3_O_4_-to-3Fe mass ratio is about 1.38, which is very close to the ratio reported in Table 4. This is a satisfactory result considering the small number of samples tested. Accurate calibration of the presented measurement system with the coil and impedance analyzer requires testing a larger number of samples on several different ICP-AES spectrometers.

**Table 5:** Atomic masses of the constituent elements of compound Fe_3_O_4_ and mass ratio of Fe_3_O_4_ to 3Fe.

Mass of Fe Atom [34]	Mass of O Atom [34]	Mass of Fe_3_O_4_ Molecule	Mass of 3Fe Atoms	Fe_3_O_4_ to 3Fe Mass Ratio
55.845 u	15.999 u	231.531 u	167.535 u	≈1.38

## 5. Conclusions

The presented paper introduces a new, quick method for testing organic soils. This method is based on impedance spectroscopy of a measuring coil, with the core being the tested material. The new method allows measuring the content of ferromagnetic iron compounds in organic soil. Unlike EIS, the presented method does not use electrodes, is sensitive to magnetic forms of iron, and does not require the measuring current to flow through the tested material—in this sense, it is a non-contact method. The test tube containing the material being tested is placed inside the measuring coil. The material does not come into contact with the coil winding. This is an important element of innovation, as this technique of examining organic soils is very poorly described in the current literature. The presented research results showed that the method allows for effective measurement of Fe_3_O_4_ content in soil in the range from 0% to 8%. An important advantage of the presented method is the relative simplicity of the measurement system design. It should be noted that the Agilent 4294A impedance analyzer used supports measurements from 40 Hz to 110 MHz. However, the presented studies showed that significant impedance changes occur only in the 115 kHz to 135 kHz band. This provides the opportunity to construct a dedicated portable impedance analyzer integrated with a measuring coil. It will operate within a narrow frequency range, making it cost-effective to construct. Moreover, traditional analytical methods require sample collection and transport to the laboratory. The proposed method allows for rapid, on-site measurements. Moreover, the presented method is being tested on natural samples, unaffected by anthropogenic influences. The presented technique is useful in studying the mechanisms of formation and transformation of iron compounds in organic environments. The above studies provide an opportunity to determine the relationship between the content of ferromagnetic minerals and the electrical and magnetic properties of the material under natural conditions, undisturbed by human activity. Further studies of impedance spectroscopy in the measuring coil are planned, taking into account not only the influence of environmental parameters, but, above all, the adjustment of the coil’s sensitivity to materials with a low content of magnetic elements. In subsequent stages, this study plans to expand to include samples from areas impacted by anthropopression and industrialization. This will enable us to determine to what extent natural processes are modified by pollution and environmental changes. That approach represents a novel direction in the analysis of environmental magnetism, combining physical and chemical methods to comprehensively describe iron transformations and identify its compounds in organic environments. Future work will focus on validating the method under field conditions, including controlled studies of moisture effects, temperature stability, and long-term calibration robustness. The development of a dedicated portable impedance analyzer operating near the coil resonance frequency is expected to improve measurement stability and facilitate reliable in situ applications.

## Figures and Tables

**Table 1:** Samples of investigated materials.

Number of Samples	S1	S2	S3
Depth of sampling below the ground	74–76 cm	160–165 cm	210–224 cm
Content of the sample (mainly)	Brown peat	Silt with plant detritus	Silt with plant detritus

**Table 2:** Content of Fe_3_O_4_ in the tested soils.

Number of Samples	S1	S2	S3
Content of Fe_3_O_4_, %	3.3 (1)	2.6 (1)	1.7 (1)

**Table 3:** ICP-AES analysis results for samples S1, S2, and S3.

Sample S1		Sample S2		Sample S3	
Element	Content, wt.%	Element	Content, wt.%	Element	Content, wt.%
Al	3.59	Al	3.91	Al	3.21
Ba	0.04	Ba	0.05		
Ca	0.19	Ca	0.29	Ca	0.15
Cr	0.01	Cr	0.01		
Fe	2.44	Fe	2.04	Fe	1.32
Mg	0.30	Mg	0.32	Mg	0.23
Mn	0.06	Mn	0.15	Mn	0.01
Ti	0.28	Ti	0.33	Ti	0.19
Zr	0.03	Zr	0.04	Zr	0.02
	**Content,** **wt. × 10^−4^%**		**Content,** **wt. × 10^−4^%**		**Content,** **wt. × 10^−4^%**
Zn	92.5	Zn	89.2	Zn	99.1
Cu	75.4	Cu	10.5	Cu	55.2
As	78.5	As	22.5	As	14.2
Ni	77.2	Ni	34.1	Ni	89.2
				Cr	55.3
				Ba	18.3

**Table 4:** Comparison of the content of Fe_3_O_4_ obtained by impedance spectroscopy and content obtained by ICP-AES.

Number of Samples	Fe_3_O_4_ Content, wt. % Result Obtained by Impedance Spectroscopy	Fe Content, wt. % Result Obtained by ICP-AES	Ratio of Contents
S1	3.3	2.44	1.35
S2	2.6	2.04	1.27
S3	1.7	1.32	1.29

## Data Availability

The original contributions presented in the study are included in the article. Further inquiries can be directed to the corresponding author.

## References

[1] Thompson R., Oldfield F. (1986). Environmental Magnetism.

[2] Shirzaditabar F., Heck R.J. (2021). Characterization of soil magnetic susceptibility: A review of fundamental concepts, instrumentation, and applications. Can. J. Soil Sci..

[3] Franciškovic-Bilinski S., Peco J., Sakan S., Dordevi D., Indi D. (2023). Magnetic and Geochemical Properties of Zagreb City Area Soils. Minerals.

[4] Shirzaditabar F., Heck R.J. (2021). Characterization of soil drainage using electromagnetic induction measurement of soil magnetic susceptibility. Catena.

[5] Porkodi G., Ramamoorthi P., David Israel Mansingh M. (2023). Effects of Iron on Crops and Availability of Iron in Soil: A Review. Biol. Forum-Int. J..

[6] Sevik H., Ozel H.U., Yildiz H., Ozel H.B. (2025). Effect of adding Fe_2_O_3_ and Fe_3_O_4_ nanoparticles to soil on germination and seedling characteristics of oriental beech. BioReources.

[7] Cooray P.L.I.G.M., Chalmers G., Chittleborough D. (2025). A review of properties of organic matter fractions in soils of mangrove wetlands: Implications for carbon storage. Soil Biol. Biochem..

[8] Huang X., Liu X., Chen L., Wang Y., Chen H. (2023). Iron-bound organic carbon dynamics in peatland profiles: The preservation equivalence of deep and surface soil. Fundam. Res..

[9] Temmink R.J.M., Lång K., Vroom R.J.E., Leifeld J., Fritz C., Zeug W., Thrän D., Kleinspehn C., Gaudig G., Neubert J. (2026). Agriculture on wet peatlands: The sustainability potential of paludiculture. Agric. Syst..

[10] Chaddha G., Seehra M.S. (2000). Magnetic components and particle size of coal flyash. J. Phys. D Appl. Phys..

[11] Hanesch M., Scholger R. (2002). Mapping of heavy metal loadings in soils by means of magnetic susceptibility measurements. Environ. Geol..

[12] Förster F. (1981). Theoretische und experimentelle Ergebnisse des magnetischen Streuflusseverfahrens. Materialprüfung.

[13] Förster F. (1986). New findings in the field of non-destructive magnetic leakage field inspection. NDT Int..

[14] Bozorth R.M. (1951). Ferromagnetism. The Institute of Electrical and Electronics Engineers.

[15] Żurek Z.H. (2005). Magnetic contactless detection of stress distribution and assembly defects in constructional steel element. NDT E Int..

[16] Żurek Z.H. (2006). Magnetic monitoring of fatigue process of the rim material of railway wheel sets. NDT E Int..

[17] Żurek Z., Chmiela B., Jasiński T., Solecka B. (2023). Validation of the impedance spectroscopy method (MSID2/3) for NDT and SHM research in the power industry. e-J. Nondestruct. Test..

[18] Clemens C., Radschun M., Jobst A., Himmel J., Kanoun O. (2021). Detection of Density Changes in Soils with Impedance Spectroscopy. Appl. Sci..

[19] Narayanan Dhamu V., Baby D., Eldeeb M., Muthukumar S., Prasa S. (2024). In-SITE: In situ soil topological examination platform for hydration state, volumetric density and carbon stocks assessment. Biosens. Bioelectron. X.

[20] Drvarič Talian S., Brutti S., Assunta Navarra M., Moškon J., Gaberscek M. (2024). Impedance spectroscopy applied to lithium battery materials: Good practices in measurements and analyses. Energy Storage Mater..

[21] Yang K., Pang Z., Song Z., Wang S., Li W., Meng J. (2024). Investigation of lead-acid battery water loss by in-situ electrochemical impedance spectroscopy. Electrochim. Acta.

[22] Yeow T., Sun J., Yao Z., Jaubert J.N., Musselman K.P. (2019). Evaluation of impedance spectroscopy as a tool to characterize degradation mechanisms in silicon photovoltaics. Sol. Energy.

[23] Katayama N., Osawa S., Matsumoto S., Nakano T., Sugiyama M. (2019). Degradation and fault diagnosis of photovoltaic cells using impedance spectroscopy. Sol. Energy Mater. Sol. Cells.

[24] Silva L.M., Martins E.C., Wulff N.A., Benedetti A.V., Hideko Y. (2025). Electrochemical Impedance Spectroscopy as a Tool to Investigate the Electrochemical Interface in Detection of Huanglongbing Bacterium Using a Magnetic Nanoparticle-Based Immunosensor. Electrochim. Acta.

[25] Pitropakis I., Pfeiffer H., Wevers M. (2012). Crack detection in aluminium plates for aerospace applications by electromagnetic impedance spectroscopy using flat coil sensors. Sens. Actuators A Phys..

[26] Magar H.S., Hassan R.Y.A., Mulchandani A. (2021). Electrochemical Impedance Spectroscopy (EIS): Principles, Construction, and Biosensing Applications. Sensors.

[27] Lasia A. (2014). Electrochemical Impedance Spectroscopy and Its Applications.

[28] Barsoukov E., Macdonald J.R. (2005). Impedance Spectroscopy Theory, Experiment, and Applications.

[29] Paul A., Eldeeb M.A., Dhamu V.N., Sharma A., Bohri S.M., Muthukumar S., Prasad S. (2025). Quantitation of total soil carbon (TSC) using an electrochemical impedance probe. Meas. Sens..

[30] Dhamu V.N., Paul A., Muthukumar S., Prasad S. (2024). Electrochemical framework for dynamic tracking of Soil Organic Matter. Biosens. Bioelectron. X.

[31] Dhamu V.N., Eldeeb M.A., Somenahally A.C., Muthukumar S., Prasad S. (2025). SODS: Soil Health On-Demand Sensors—A Multi Parameter Field Study with Temporal Monitoring. Sensors.

[32] (2020). Impedance Measurement Handbook—A Guide to Measurement Technology and Techniques.

[33] Solecka B., Tytko G., Michczyński A., Feng B., Xie Y. (2025). Metal powder testing with the employment of electrical impedance spectroscopy. Measurement.

[34] Encyclopedia Britannica. https://www.britannica.com/.

